# Inhibition of peripheral chemoreceptors improves ventilatory efficiency during exercise in heart failure with preserved ejection fraction − a role of tonic activity and acute reflex response

**DOI:** 10.3389/fphys.2022.911636

**Published:** 2022-08-30

**Authors:** Katarzyna Kulej-Lyko, Piotr Niewinski, Stanislaw Tubek, Magdalena Krawczyk, Wojciech Kosmala, Piotr Ponikowski

**Affiliations:** ^1^ Institute of Heart Diseases, Wroclaw Medical University, Wroclaw, Poland; ^2^ Department of Cardiology, University Clinical Hospital, Wroclaw, Poland

**Keywords:** peripheral chemosensitivity, heart failure with preserved ejection fraction, exercise tolerance, tonic activity, reflex response

## Abstract

Peripheral chemoreceptors (PChRs) play a significant role in maintaining adequate oxygenation in the bloodstream. PChRs functionality comprises two components: tonic activity (PChT) which regulates ventilation during normoxia and acute reflex response (peripheral chemosensitivity, PChS), which increases ventilation following a specific stimulus. There is a clear link between augmented PChS and exercise intolerance in patients with heart failure with reduced ejection fraction. It has been also shown that inhibition of PChRs leads to the improvement in exercise capacity. However, it has not been established yet: 1) whether similar mechanisms take part in heart failure with preserved ejection fraction (HFpEF) and 2) which component of PChRs functionality (PChT vs. PChS) is responsible for the benefit seen after the acute experimental blockade. To answer those questions we enrolled 12 stable patients with HFpEF. All participants underwent an assessment of PChT (attenuation of minute ventilation in response to low-dose dopamine infusion), PChS (enhancement of minute ventilation in response to hypoxia) and a symptom-limited cardiopulmonary exercise test on cycle ergometer. All tests were placebo-controlled, double-blinded and performed in a randomized order. Under resting conditions and at normoxia dopamine attenuated minute ventilation and systemic vascular resistance (*p* = 0.03 for both). These changes were not seen with placebo. Dopamine also decreased ventilatory and mean arterial pressure responses to hypoxia (*p* < 0.05 for both). Inhibition of PChRs led to a decrease in V˙E/V˙CO_2_ comparing to placebo (36 ± 3.6 vs. 34.3 ± 3.7, *p* = 0.04), with no effect on peak oxygen consumption. We found a significant relationship between PChT and the relative decrement of V˙E/V˙CO_2_ on dopamine comparing to placebo (R = 0.76, *p* = 0.005). There was a trend for correlation between PChS (on placebo) and V˙E/V˙CO_2_ during placebo infusion (R = 0.56, *p* = 0.059), but the relative improvement in V˙E/V˙CO_2_ was not related to the change in PChS (dopamine vs. placebo). We did not find a significant relationship between PChT and PChS. In conclusion, inhibition of PChRs in HFpEF population improves ventilatory efficiency during exercise. Increased PChS is associated with worse (higher) V˙E/V˙CO_2_, whereas PChT predicts an improvement in V˙E/V˙CO_2_ after PChRs inhibition. This results may be meaningful for patient selection in further clinical trials involving PChRs modulation.

## Introduction

Heart failure (HF) became a true epidemy of 21^st^ century with the prevalence of 1-2% (Groenewegen et al., 2020) in developed countries. At least half of HF patients is characterized by preserved ejection fraction of left ventricle (heart failure with preserved ejection fraction, HFpEF) (Bursi et al., 2006). The number of patients with HFpEF is constantly rising (in contrast to patients with HF with reduced ejection fraction, HFrEF) leading to significant increment in hospitalization rates and costly burden on the health care systems (Owan et al., 2006; Steinberg et al., 2012; Tsao et al., 2018).

Clinically HFpEF presents with typical signs and symptoms of HF (McDonagh et al., 2021). Among them exercise intolerance seems to be most debilitating, affecting normal daily activities and being linked to worse outcomes (Owan et al., 2006; Tsao et al., 2018). The mechanism underlying exercise intolerance in HFpEF population is complex and not clearly understood. Previous studies have suggested various explanations for this phenomenon including: abnormal left-ventricle-central vascular coupling (Kosmala et al., 2016), increased left ventricular filling pressure during exercise (Borlaug et al., 2010), chronotropic incompetence (Borlaug et al., 2006), impaired left ventricular systolic and diastolic reserve (Kosmala et al., 2016; Kosmala, Przewlocka-Kosmala and Marwick, 2019), diminished cardiac output on exercise (Abudiab et al., 2013) and blunted oxygen extraction in the periphery (due to microvascular dysfunction and/or intrinsic muscle abnormalities) (Dhakal et al., 2015).

There is some evidence that abnormal reflex control contributes to exercise intolerance in HFrEF (Ponikowski et al., 1997, 2001). This includes an enhanced reflex response from peripheral chemoreceptors (PChRs) (Chua, Ponikowski et al., 1996, 1997). PChRs are multimodal sensors located mainly within the carotid bodies (CBs) close to the bifurcation of the common carotid artery but also within the structures, known as the aortic bodies (ABs), along the aortic arch (Paton, Sobotka et al., 2013). Apart from the ability to detect hypoxemia (O’Regan and Majcherczyk, 1982), PChRs are sensitive to various stimuli including: carbon dioxide (Nye, 1994), hydrogen ions (Nurse, 2009), glucose (Allen, 1998), lactate (Torres-Torrelo et al., 2021), potassium (McLoughlin, Linton and Band, 1995) and angiotensin II (Allen, 1998). Some of these stimuli may be in play during exercise and thus activate PChRs. Excitation of CBs provokes stimulation of the nuclei of the solitary tract with subsequent activation of the paraventricular nucleus of the hypothalamus, pre-Botzinger complex (Ramirez et al., 1998) and pre-sympathetic neurons localized in rostral ventrolateral medulla (RVLM) (Chen, Du and Wang, 2004; Reddy, Patel and Schultz, 2005; Schultz, Li and Ding, 2007). This results in augmentation of the minute ventilation (Ponikowski and Banasiak, 2001), activation of the sympathetic drive (leading to an increase in blood pressure), tachycardia (through the activation of Hering-Breuer reflex and due to direct stimulation of ABs) (Niewinski and Janczak, 2014) and decrease in the barosensitivity (Despas et al., 2012).

Apart from an augmented reflex response (acute sensitivity, PChS), the potential role of heightened tonic activity (tonicity, PChT) of PChRs in exercise intolerance must be acknowledged. Its relation to worse exercise tolerance in HF population has not been extensively studied. Similarly, it is unclear whether the level of tonic activity is related to the sensitivity of acute reflex response in a given individual with HF. It is possible to directly assess the magnitude of tonic activation of PChRs using various approaches including hyperoxia (Tubek et al., 2021) and low-dose dopamine infusion (Niewinski and Tubek, 2014).

The involvement of PChRs in exercise intolerance in HF is supported by the results of experiments with oxygen and opioids (Chua, Ponikowski et al., 1996; Chua, Harrington et al., 1997) and recently by CB resection trial (Niewinski et al., 2017) in which prolongation of exercise time and decrease in slope relating ventilation to carbon dioxide (V˙E/V˙CO_2_ slope) were observed. Based on these results obtained from HFrEF patients, one could speculate, that similar mechanisms may be in play in HFpEF. Data from some animals models indicate that central but not peripheral chemoreceptors are predominantly responsible for the autonomic imbalance seen in HFpEF (Kristen et al., 2002; Toledo et al., 2017). On the contrary, augmented PChS is often encountered in patients with metabolic disorders (Cunha-Guimaraes et al., 2020) and chronic obstructive pulmonary disease (Stickland et al., 2016), while exaggerated PChT has been reported in hypertension (Sinski et al., 2014). The above diseases commonly coexist with HFpEF.

We hypothesize, that PChRs play an important role in exercise intolerance in HFpEF population. In order to verify this hypothesis we designed this double-blinded, placebo controlled experiment. The low-dose dopamine infusion, which is known to diminish both PChS and PChT (van de Borne, Oren and Somers, 1998), or placebo were administered during incremental cardiometabolic stress in HFpEF patients. Additionally, to determine which component of PChRs functionality is responsible for exercise intolerance, the separate measurements of individual PChS (employing intermittent hypoxia method) and PChT (expressed as the magnitude of the change in minute ventilation following low-dose dopamine infusion initiation) were performed in resting conditions and compared with the results of cardiometabolic stress tests.

## Methods

### Studied population

Twelve patients (three men, nine women) with a diagnosis of HFpEF in NYHA (New York Heart Association) II or III functional class were enrolled into the study after signing an informed consent. All subjects had to be clinically stable and optimally treated for at least 12 weeks before the study entry. The diagnosis of HFpEF, according to the current European Society of Cardiology guidelines criteria, was verified at initial evaluation and was based on the presence of signs and symptoms of HF, results of blood sampling, spirometry, electrocardiography and echocardiography (Ponikowski et al., 2016; McDonagh et al., 2021). The presence of a severe untreated or treated valvular heart disease, severe pulmonary hypertension, symptomatic asthma or chronic obstructive pulmonary disease, uncontrolled hypertension, severe renal impairment (estimated glomerular filtration rate (eGFR) < 30 ml/min/1.73 m^2^ using Cockcroft-Gault formula) (Cockcroft and Gault, 1976), serious anaemia, or severe physical disability precluding exercise test on cycle ergometer were considered as exclusion criteria.

The study was performed in accordance with the Helsinki Declaration and was approved by the local Institutional Ethics Committee (The Bioethics Committee at the Wroclaw Medical University).

### Study protocol

Following initial evaluation all patients filled in the quality of life questionnaires, namely Minnesota Living With Heart Failure Questionnaire (MLWHFQ) (Rector, Kubo and Cohn, 1987; Garin et al., 2014) and Kansas City Cardiomyopathy Questionnaire (KCCQ) (Green et al., 2000; Joseph et al., 2013) and underwent exercise capacity assessment with 6-min walking test. During subsequent visits at an interval of 1-7 days patients were subjected to: 1) testing of the function of PChRs during low-dose dopamine infusion, 2) testing of the function of PChRs during placebo infusion, 3) symptom-limited cardiopulmonary exercise test during low-dose dopamine infusion and 4) symptom-limited cardiopulmonary exercise test during placebo infusion. Tests were performed in randomized, double-blinded fashion in a silent, air-conditioned room at an ambient temperature of 22°C. During one visit no more than one test of each type took place. The solutions were administered through the venous catheter placed into cephalic vein with the use of an infusion pump (Perfusor Space, B. Braun, Germany). Dopamine was administered at the dose of 3 μg kg^−1^ min^−1^, which is known to effectively inhibit PChRs (Horwitz, Fox and Goldberg, 1962; Heistad et al., 1972). The same volume of normal saline solution was used as a placebo. Before each test patients were asked to take their usual medications in the morning, stay fasting and avoid caffeine or nicotine intake for at least 12 h prior to the visit.

### Echocardiography

Echocardiographic imaging was performed according to recommendations of the American Society of Echocardiography and the European Association of Cardiovascular Imaging (Lang et al., 2015; Nagueh et al., 2016) using standard equipment (Vivid S70N, General Electric Medical Systems, Milwaukee, Wisconsin).

### Assessment of peripheral chemoreflex

Peripheral chemoreceptors function testing was performed twice in each subject—during an infusion of low-dose dopamine and during the placebo infusion in a supine position. After adjusting silicone nasofacial mask (Hans Rudolph, Inc, Shawnee, Kansas) patients were connected to a one-way open breathing circuit. A spirometry set (flowhead—MTL3000L, and pressure transducer—FE141 Spirometer, ADInstruments, Sydney, Australia) was attached to the expiratory arm of the circuit for continuous measurements of tidal volume (TV) and breathing rate (BR) from which minute ventilation (MV) was calculated. End-tidal CO_2_ (EtCO_2_) was sampled from the expiratory arm of the circuit and measured with capnograph (Capstar 100; CWE, Ardmore, Pennsylvania). The inspiratory arm of the circuit was connected to the electric valve allowing for the silent switching between 100% nitrogen gas and room air. Hemodynamic parameters including: mean blood pressure (MAP), heart rate (HR), cardiac output (CO) and systemic vascular resistance (SVR) were continuously, non-invasively measured using Nexfin device (BMEYE B.V. Amsterdam, Netherlands). Oxygen saturation (SpO_2_) was collected with a pulse oximeter (Radical-7; Masimo Corp., Irvine, CA, United States) using a lightweight probe attached to the earlobe. All the parameters were processed with PowerLab (ADInstruments, Sydney, Australia) and recorded on a laptop computer (Dell Inc., Round Rock, TX, United States).

**FIGURE 1:**
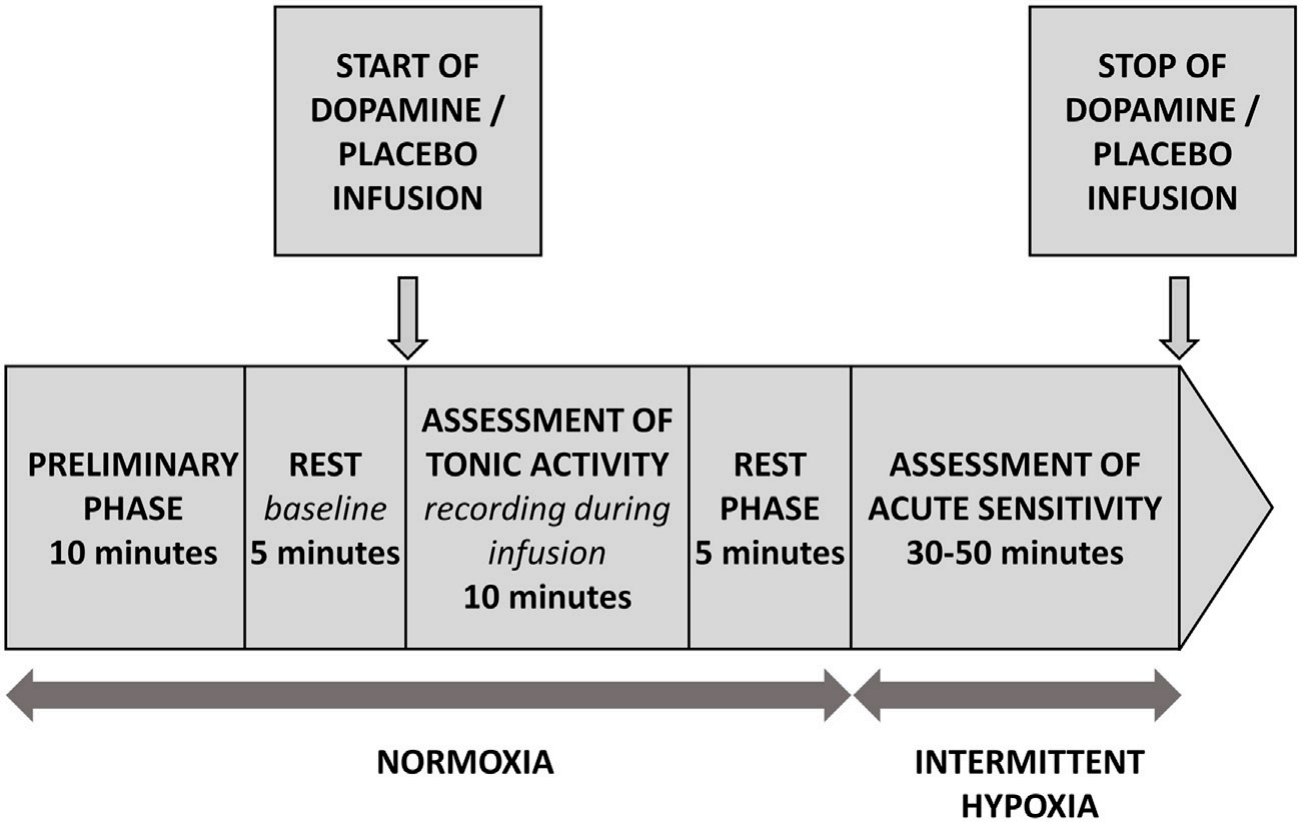
Study protocol.

All patients were tested according to the same protocol depicted on Figure 1. Briefly, following the first 10 min necessary for the subject`s familiarization with the study environment (not included into the analysis), 5 min of *baseline* was recorded. Subsequently, the infusion of dopamine or placebo was initiated and continued for 10 min. This period was defined as “recording during infusion”. Next, after 5 min of rest, an analysis of acute sensitivity commenced which consisted of several (5-7) administrations of pure nitrogen gas (each for 2-20 s), which resulted in minimal values of SpO_2_ in a range between 90% and 65%. Length and order of hypoxic exposures was random and they were separated by 3-4 min of breathing with room air which allowed for the normalization of recorded parameters. Finally, the infusion of dopamine or placebo was withdrawn and the test ended.

### Assessment of PChS and hemodynamic response to hypoxia

**FIGURE 2:**
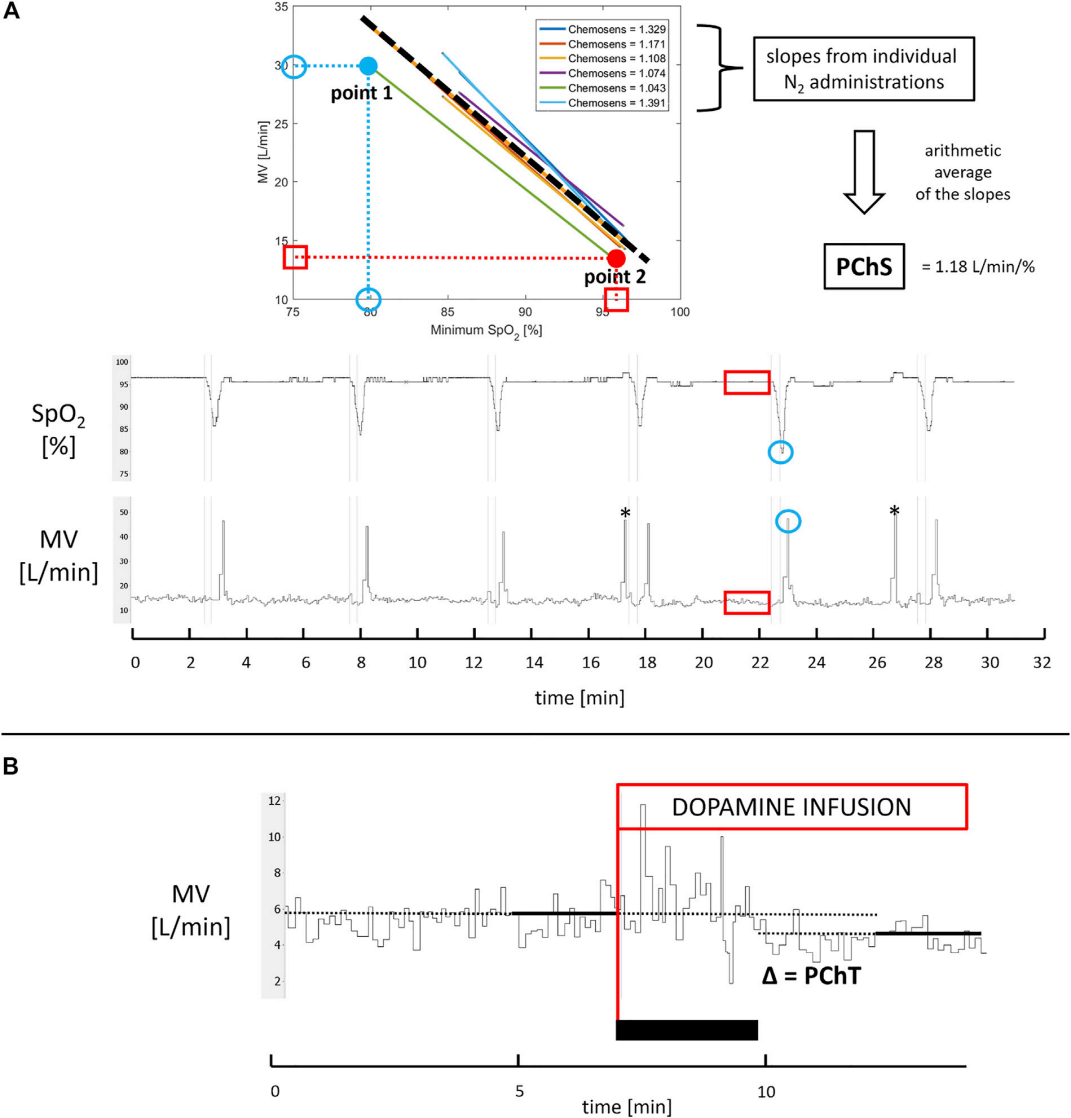
Assessment of PChS and PChT. Panel **(A)** For each N_2_ administration (thin vertical lines marking start and stop of N_2_ exposures) minimal SpO_2_ and corresponding maximal minute ventilation (MV) averaged from three consecutive breaths were identified (blue circles; note that MV tracing is recorded breath-by-breath) to provide point 1. Next, 90 s recordings of SpO_2_ and MV directly preceding N_2_ administration were averaged to provide point 2 (red squares and rectangles). The slope of regression function linking point 1 and point 2 was calculated for each N_2_ exposure. The average of all slopes (dashed thick line) constitutes PChS. * indicates incidental gasps ignored in PChS assessment. Panel **(B)** PChT was calculated as an absolute difference between MV averaged from 2 min directly preceding initiation of low-dose dopamine infusion (red line) and MV obtained from 2 min of recording during dopamine infusion when ventilatory and hemodynamic parameters stabilized (the black rectangle indicates stabilization phase when dopamine does not exert its full action yet).

PChS was assessed using well-described transient hypoxia method (Rebuck and Campbell, 1974; Chua and Coats, 1995). Subjects were silently switched from breathing room air to breathing N_2_ gas for time period lasting between 2 s and 20 s. This procedure was repeated 5–7 times per study participant which resulted in minimal SpO_2_ between 90% and 65%. After each N_2_ administration subjects were allowed to rest for 5 min breathing room air. A ventilatory response was averaged from the three largest consecutive breaths following the end of N_2_ administration and plotted against the associated nadir of SpO_2_ providing Point 1. Baseline values of MV and SpO_2_ were averaged from a 90 s period preceding each N_2_ administration. Then, baseline MV was plotted against baseline SpO_2_ providing Point 2. The slope of the regression line linking Point 1 and Point 2 was found for each N_2_ exposure. Arithmetic average of values of the slopes for all N_2_ administrations (Prasad et al., 2020) was taken as a measure of PChS (l min^−1^ %^−1^) (Figure 2A).

The hemodynamic responses to hypoxia were calculated in the analogous way as PChS. Hemodynamic data were smoothed by a moving average (10 s window) in order to eliminate the effect of fluctuations in HR caused by respiratory sinus arrythmia or atrial fibrillation. The slope of linear regression describing the relation between the peak values of HR and MAP after nitrogen administrations and corresponding SpO_2_ nadirs was calculated (HR slope and MAP slope respectively) to express the magnitude of acute hypoxic reactivity of a given hemodynamic variable.

### Assessment of PChT

PChT (l min^−1^) was defined as the absolute decrease in MV following initiation of dopamine infusion. MV was averaged from the last 2 min of steady *baseline* recording and from the 2 min of *recording during infusion*, when changes in ventilatory and hemodynamic parameters stabilized following initiation of dopamine infusion (Figure 1 and Figure 2B).

### Assessment of exercise capacity

Symptom-limited cardiopulmonary exercise test using cycle ergometer (Ergoselect 5, Ergoline GmbH, Germany) was performed in the upright seated position twice in each subject—during an infusion of low-dose dopamine and on saline. During the tests ventilatory parameters, end-tidal CO_2_ and end-tidal oxygen were collected with pneumotachometer (Ergoflow, Reynolds Medical, Poland). ECG was continuously monitored during the stress test (Amedtec ECG, Reynolds Medical, Poland). The cardiopulmonary exercise tests begun with a 5-min long resting phase to allow for familiarization with the equipment. Following the resting phase workload was set at 20 W and increased by 10 W every minute until maximal tolerated level of exertion was achieved (Borlaug et al., 2011). The subjects were instructed to pedal with a constant cadence of 60 ± 5 revolutions per minute (rpm). Exercise phase was followed by a 5-min long recovery phase.

The following variables were automatically collected and calculated from the last 30 s of the cardiopulmonary exercise test (Blue Cherry, Reynolds Medical, Poland): peak oxygen uptake (peak V˙O_2_), peak carbon dioxide production (peak V˙CO_2_), slope relating ventilation to oxygen uptake (V˙E/V˙O_2_ slope), slope relating ventilation to carbon dioxide production (V˙E/V˙CO_2_ slope), respiratory exchange ratio (RER) (Glaab and Taube, 2022), and peak workload (Watts). We also recorded total exercise time (s) and reported V˙E/V˙CO_2_ nadir defined as the lowest V˙E/V˙CO_2_ before the respiratory compensation point. Nadir V˙E/V˙CO_2_ is believed to be the most accurate tool in estimation of ventilatory efficiency because it is independent of exercise workload, hyperventilation and metabolic acidosis at peak exercise (Whipp and Ward, 1982; Phillips, Collins and Stickland, 2020).

### Data and statistical analysis

LabChart 8 (ADInstruments, Sydney, Australia), MATLAB (MathWorks, Natick, MA, United States) and Statistica 13 (StatSoft Inc., Tulsa, OK, United States) were used for analyzing the data. The data were blinded for the researchers responsible for PChS and PChT calculations. The statistical analysis was performed with the Wilcoxon matched pairs test and variables were presented as mean and standard deviation (SD) or mean and standard error of the mean (SEM). Spearman rank method was used for correlation calculation. *P* value < 0.05 was assumed as statistically significant.

## Results

### Baseline characteristics of the study population

The baseline clinical and physiological characteristics of the study population (n = 12) are presented in Table 1 and Table 2 respectively. We did not find differences between baseline hemodynamic and ventilatory parameters recorded before dopamine and placebo infusions.

**TABLE 1:** Clinical characteristics of the study population.

Clinical variables	
Age [years]	73 ± 7
Gender [female/male]	9/3
BMI [kg/m^2^]	32.2 ± 6
NYHA class (II/III) [%]	83.3/16.7
Smoking [yes, %]	8
6MWT [m]	398 ± 129
Peak V^·^O_2_ [ml kg^−1^min^−1^]	14.8 ± 3.4
MLHFQ [points]	35 ± 23
KCCQ [points]	88 ± 21
Echocardiographic parameters	
LVEF [%]	58 ± 3
LVMI [g m^−2^]	116 ± 29
Left ventricular hypertrophy [%]	75
LAVI [ml m^−2^]	49 ± 22
E/e’	14 ± 3
TRPG [mmHg]	26 ± 6
Spirometry	
FEV_1_/FVC [%]	105 ± 12
Blood tests measurements	
NTproBNP [pg/mL]	712 ± 688
Haemoglobin [g/dL]	13.3 ± 1.3
eGFR ml/min/1.73 m^2^	70.1 ± 28.2
Comorbidities	
Hypertension [%]	100
Atrial fibrillation [%]	50
Ischaemic heart disease [%]	33.3
Dyslipidemia [%]	83.3
Diabetes mellitus [%]	25
Chronic kidney disease defined as eGFR <60 ml min/1.73 m^2^ [%]	50
Treatment	
Loop diuretics [%]	50
Thiazide diuretics [%]	17
Βeta-blockers [%]	92
ACEI/ARB [%]	92
Aldosterone antagonists [%]	33

Values are presented as mean ± SD.

BMI- body mass index, NYHA- New York Heart Association, 6MWT- 6-minutes walking test, peak V^·^O_2_- peak oxygen consumption, MLHFQ- Minnesota Living With Heart Failure Questionnaire, KCCQ- The Kansas City Cardiomyopathy Questionnaire, LVEF- left ventricular ejection fraction, LVMI- left ventricular mass index, LAVI- left atrial volume index, E/e’- ratio of mitral peak velocity of early filling (E) to early averaged diastolic mitral annular velocity (e’), TRPG- tricuspid regurgitation peak gradient, FEV_1_/FVC (Tiffeneau index)- ratio between forced expiration in the first second (FEV_1_) and forced vital capacity (FVC), NTproBNP- N-terminal pro-B type natriuretic peptide, eGFR- estimated glomerular filtration rate with Cockcroft-Gault formula, ACEI- angiotensin-converting enzyme inhibitors, ARB- angiotensin receptor blockers.

**TABLE 2:** Baseline ventilatory and hemodynamic indices.

Baseline ventilatory and hemodynamic parameters	Pre- placebo infusion	Pre- dopamine infusion
BR [breaths min^−1^]	16 ± 4	17 ± 4
MV [l min^−1^]	9.6 ± 3.2	10 ± 4
TV [l]	0.6 ± 0.2	0.6 ± 0.3
EtCO_2_ [mmHg]	38.2 ± 3.7	37.1 ± 3.5
SpO_2_ [%]	95 ± 2	95 ± 2
SVR [dyn s cm^−5^]	1578 ± 667	1429 ± 406
HR [beats minute^−1^]	60 ± 5	62 ± 5
CO [l min^−1^]	4.8 ± 1.2	5.0 ± 1.1
MAP [mmHg]	82 ± 12	81 ± 8

Values are presented as mean ± SD.

All p = not significant.

BR- breathing rate, MV- minute ventilation, TV- tidal volume, EtCO_2_- end-tidal carbon dioxide, SpO_2_- oxygen saturation, SVR- systemic vascular resistance, HR- heart rate, CO- cardiac output, MAP- mean arterial pressure.

### Assessment of PChT

**FIGURE 3:**
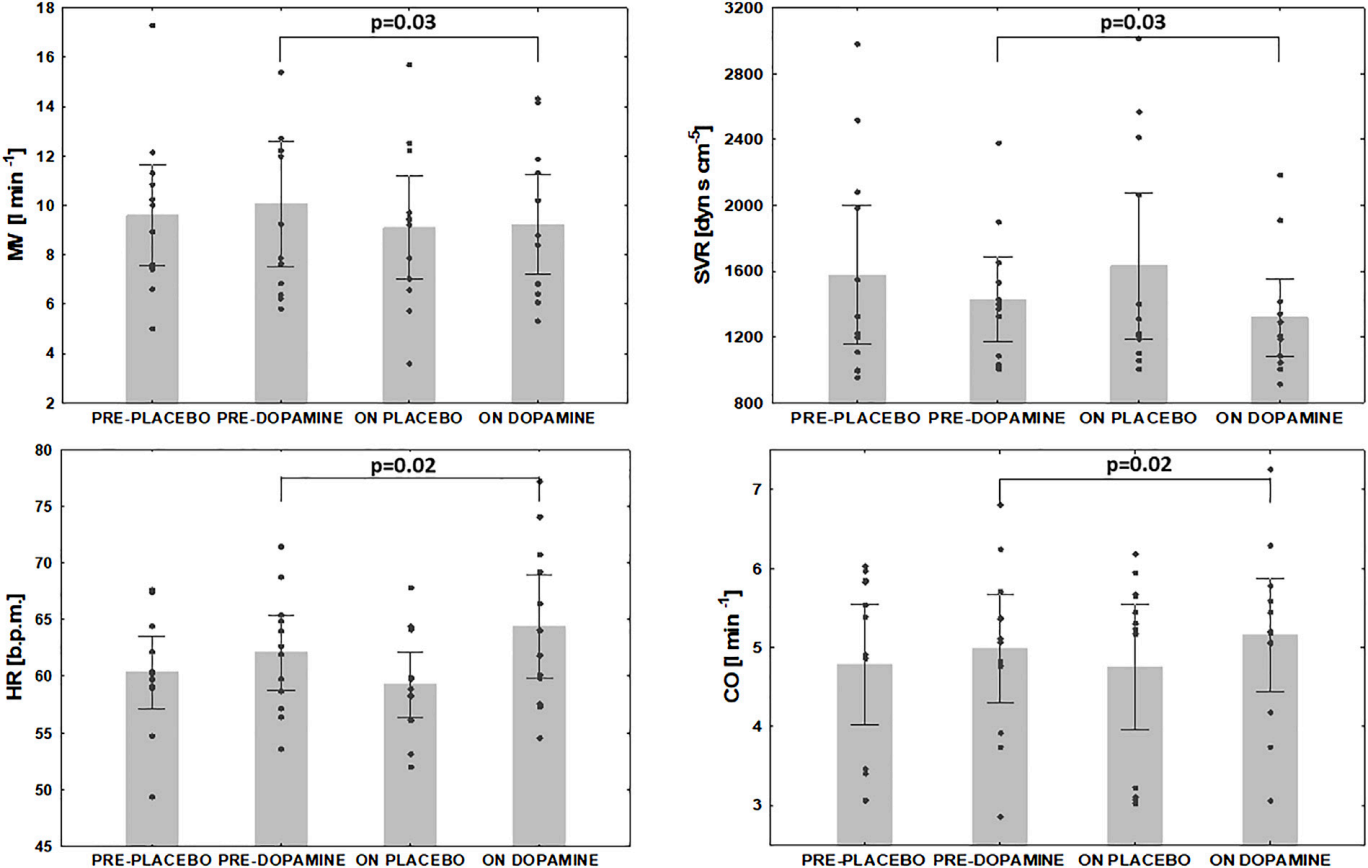
The effect of dopamine infusion on ventilation and hemodynamic indices under normoxia. MV- minute ventilation, SVR- systemic vascular resistance, HR- heart rate, CO- cardiac output.

Under normoxic resting conditions dopamine administration significantly attenuated MV (10.0 ± 4.0 vs. 9.2 ± 3.1 L/min, *Δ* = 6.2%, *p* = 0.03), SpO_2_ (95 ± 2 vs. 93 ± 3%, *Δ* = 1.8%, *p* = 0.005), SVR (1429 ± 406 vs. 1318 ± 375 dyn s cm^−5^, *Δ* = 7.3% *p* = 0.03) but increased HR (62 ± 5 vs. 64 ± 7 beats per minute, *Δ* = 3.6%, *p* = 0.02) and CO (5.0 ± 1.1 vs. 5.2 ± 1.1 L min^−1^, *Δ* = 3.6%, *p* = 0.02) (Figure 3). We also noted a trend for decrease in MAP after initiation of dopamine (81 ± 8 vs. 78 ± 7 mmHg, *Δ* = 3.6%, *p* = 0.07) and a trend for increase in EtCO_2_ (37.1 ± 3.5 vs. 38.1 ± 3.8 mmHg, *Δ* = 2.7%, *p* = 0.07). After placebo infusion a fall in SpO_2_ (95 ± 2 vs. 94 ± 3%, *Δ* = 1.2%, *p* = 0.02) and increase in MAP (82 ± 12 mmHg vs. 84 ± 11 mmHg, *Δ* = 2.2%, *p* = 0.03) were observed while other ventilatory and hemodynamic parameters remained unaffected.

### Assessment of PChS on/off dopamine

**FIGURE 4:**
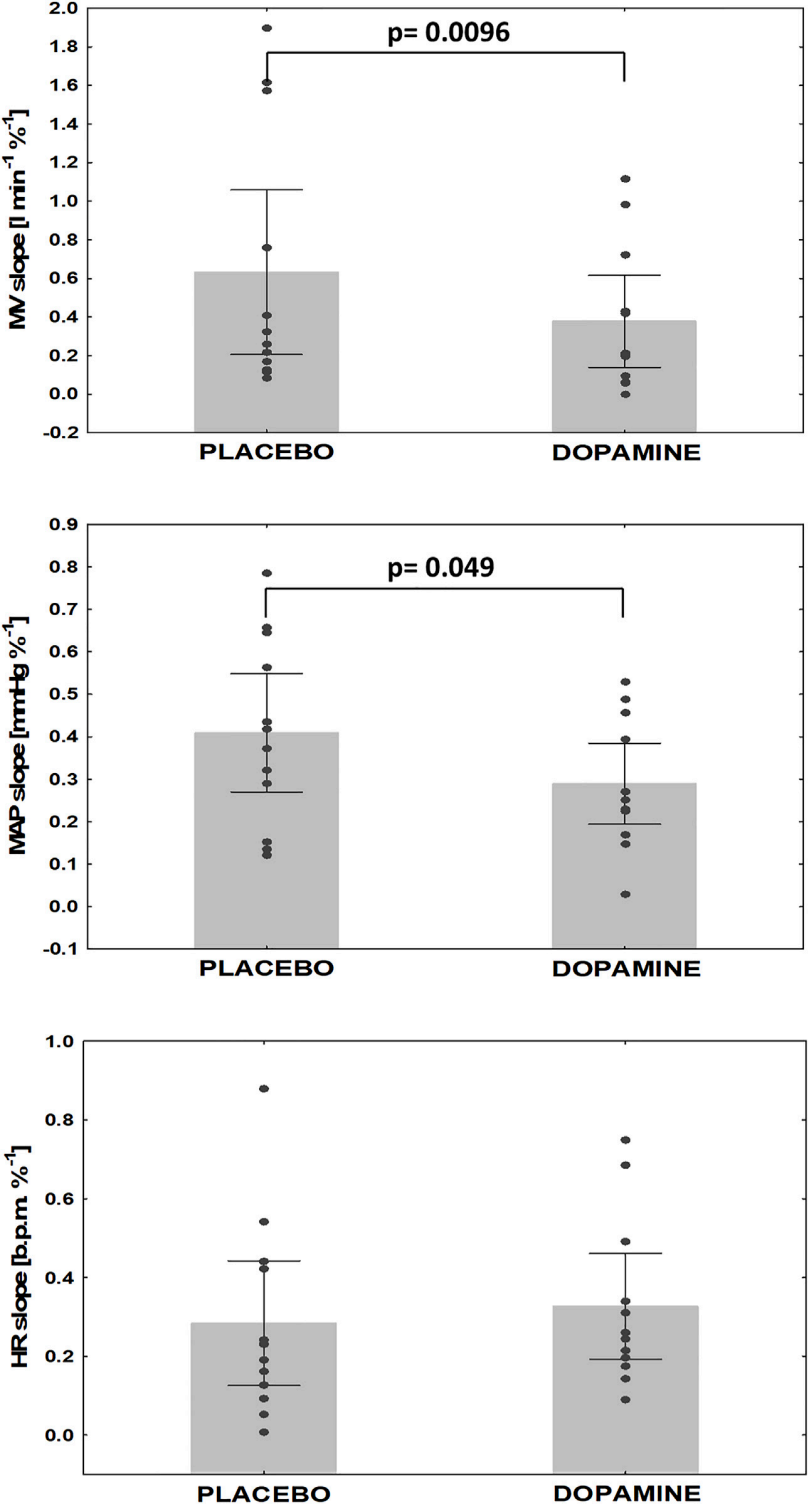
Changes in hypoxic reactivity on dopamine vs. placebo. MV- minute ventilation, MAP- mean arterial pressure, HR- heart rate.

The mean PChS measured during placebo infusion was -0.6 ± 0.7 L min^−1^ %^−1^ comparing to -0.4 ± 0.4 L min^−1^ %^−1^ (*Δ* = 35.1%) on dopamine. This difference was statistically significant (*p* = 0.0096). Additionally, dopamine infusion decreased MAP slope (-0.4 ± 0.2 vs. -0.3 ± 0.2 mmHg %^−1^, *Δ* = 10.1%, *p* = 0.049) with no effect on HR slope (-0.3 ± 0.2 vs. -0.3 ± 0.2 b.p.m %^−1^, *Δ* = 2.5%, p = NS) (Figure 4). There was a significant decrease in EtCO_2_ when baseline values were compared to minimal EtCO_2_ following nitrogen administration (38.4 ± 4.3 vs. 33.1 ± 4.6 mmHg, *Δ* = 13.8%, and 38.2 ± 4.0 vs. 33.8 ± 4.1 mmHg, *Δ* = 13.3%, for placebo and dopamine respectively; *p* = 0.005 for both).

### Relation between PChS, PChT and exercise tolerance

We did not find a relation between PChS (when measured on placebo) and PChT (*p* = 0.24). There was a trend for a correlation between PChS on placebo and V˙E/V˙CO_2_ slope on placebo (R = 0.56, *p* = 0.059). Hemodynamic slopes on placebo (HR slope, MAP slope) did not correlate with peak exercise indices (p = NS for all).

Interestingly, significant positive relation was found between PChT and exercise time (R = 0.73, *p* = 0.007) and between PChT and peak workload (R = 0.58, *p* = 0.048). No other correlations were found between PChS, PChT and exercise-derived parameters.

### The impact of PChRs inhibition on exercise tolerance

The peak exercise values of the ventilatory and hemodynamic parameters are presented in Table 3. Dopamine decreased V˙E/V˙CO_2_ slope (36 ± 3.6 vs. 34.3 ± 3.7, *p* = 0.04) and increased EtCO_2_ (33.7 ± 3.0 vs. 35.3 ± 3.8 mmHg, *p* = 0.02) but had no effect on other reported peak exercise parameters (p = NS for all). Furthermore, dopamine improved ventilatory efficiency by reducing V˙E/V˙CO_2_ nadir (32 ± 3.4 vs. 30.8 ± 3.7, *p* = 0.03).

**TABLE 3:** The effect of dopamine on peak exercise parameters.

Peak exercise parameters	Placebo	Dopamine
V˙O_2_ [ml kg^−1^ min^−1^]	14.8 ± 3.4	15.3 ± 3.9
V˙CO_2_ [l min^−1^]	1342.1 ± 491.8	1399.7 ± 634.4
V˙E/V˙O_2_	39.2 ± 3.9	37.7 ± 4.4
V˙E/V˙CO_2_	36.0 ± 3.6	34.3 ± 3.7*
TV [l]	1.52 ± 0.45	1.51 ± 0.47
BF [min^−1^]	35 ± 6	33 ± 5
EtCO_2_ [mmHg]	33.7 ± 3.0	35.3 ± 3.8*
RER	1.1 ± 0.1	1.1 ± 0.1
SBP [mmHg]	153 ± 21	153 ± 20
DBP [mmHg]	74 ± 9	74 ± 12
HR [beats minute^−1^]	106 ± 22	106 ± 16
Exercise duration [s]	498 ± 233	480 ± 223
Workload [Watt]	96.7 ± 38.9	95.8 ± 35.8

Values are presented as mean ± SD.

* *p* < 0.05 for dopamine vs. placebo.

V˙O_2_- oxygen consumption, V˙CO_2_- carbon dioxide production, V˙E/V˙O_2_- the ratio between ventilation and oxygen consumption, V˙E/V˙CO_2_- the ratio between ventilation and carbon dioxide production, TV- tidal volume, BF- breathing frequency, EtCO_2_- end-tidal CO_2_, RER- respiratory exchange ratio, SBP- systolic blood pressure, DBP-diastolic blood pressure, HR- heart rate.

**FIGURE 5:**
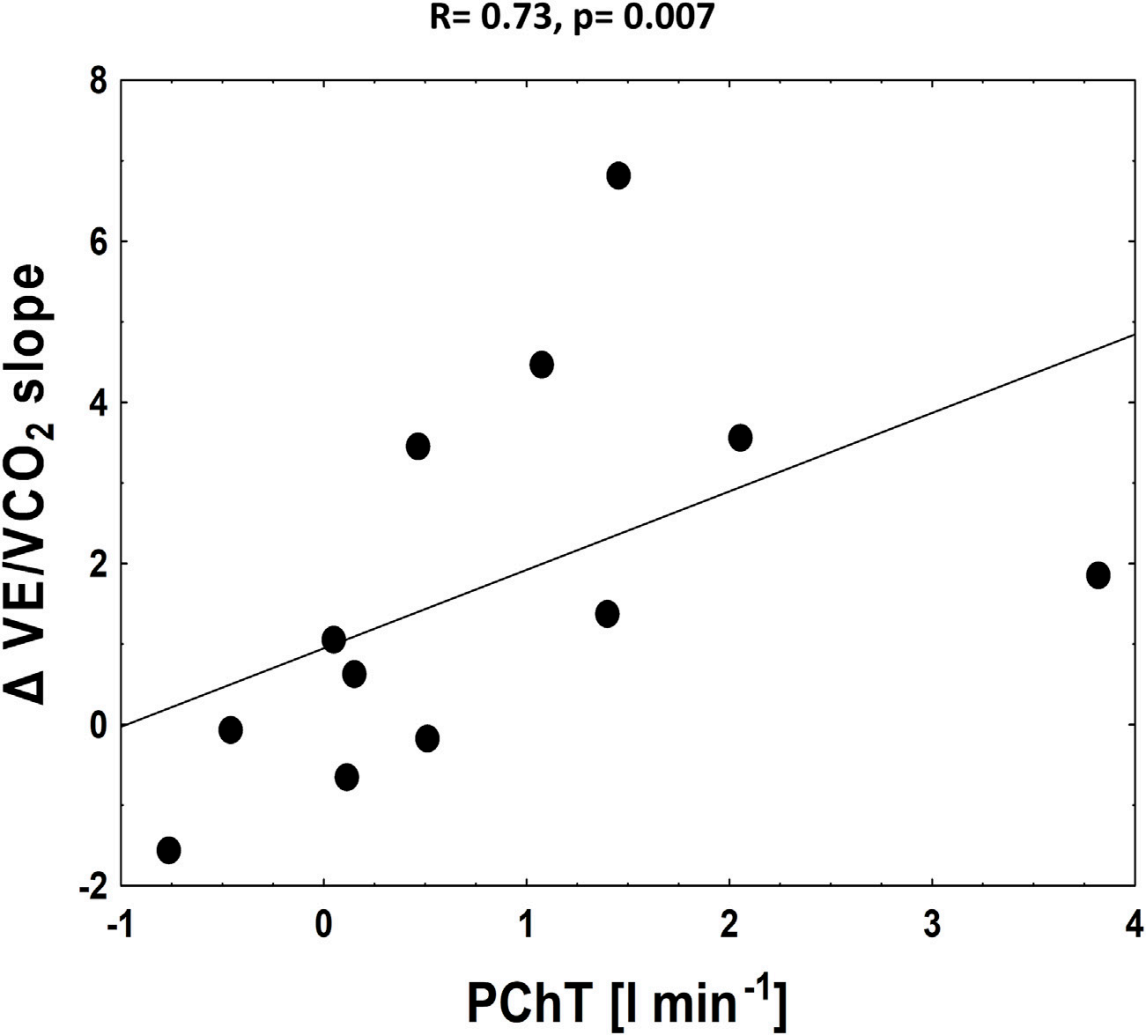
The correlation between absolute improvement in V˙E/V˙CO_2_ slope and PChT (tonic activity).

We found that both relative and absolute improvement in V˙E/V˙CO_2_ slope was related to PChT (R = 0.76, *p* = 0.005 and R = 0.73, *p* = 0.007 respectively) (Figure 5). Such correlation was found neither for PChS on placebo nor for the change in PChS (relative and absolute) following dopamine infusion (p = NS for all).

## Discussion

To the best of our knowledge, this is the first double-blinded, placebo controlled study investigating the role of PChRs in diminished exercise capacity in patients with HFpEF. The major finding of our study is the improvement in ventilatory efficiency during exercise (decrease in V˙E/V˙CO_2_ slope) following inhibition of PChRs with low-dose dopamine. We also present the inhibitory effect of dopamine on ventilatory response to hypoxia, and on resting minute ventilation. Using a novel approach based on the separate assessment of two components of PChRs functionality (Figure 2) we showed that the magnitude of this benefit depends on tonic activity of PChRs, whereas hyperreflexia constitutes a marker of poor exercise tolerance, but has no bearing on the response to the inhibitory maneuver.

### The mechanism of exercise intolerance in the HFpEF population

Exercise intolerance is a cardinal manifestation of heart failure with preserved ejection fraction. Pathogenesis of this syndrome is multifactorial and involves cardiac, pulmonary, muscular, peripheral and autonomic abnormalities (Nayor et al., 2020). The HFpEF patients, despite preserved ejection fraction of left ventricle, present significant impaired of exercise capacity similar to those with reduced ejection fraction. Chronotropic incompetence (Borlaug et al., 2006), reduced systolic and diastolic left ventricular capacity (Kosmala, Przewlocka-Kosmala and Marwick, 2019), abnormal left-ventricle systemic vascular coupling (Kosmala et al., 2016), reduced function of left atrium (Reddy et al., 2017), right ventricle and pulmonary vascular dysfunction (Del Buono et al., 2019) limit the left ventricular capacity in increasing cardiac output during exercise which provokes significant rise in pulmonary capillary arterial pressure on exertion. Another reason of reduced physical capacity in the HFpEF population is the lung dysfunction: mismatch between ventilation and perfusion (Olson, Johnson and Borlaug, 2016), unfavourable pulmonary vascular remodelling and limited lung compliance (Malhotra et al., 2016). Additionally, skeletal muscular myopathy (structural and functional) (Drexler et al., 1992; Wilson, Mancini and Dunkman, 1993) with peripheral endothelial dysfunction (Dhakal et al., 2015) disrupt oxygen utilization in the periphery and attenuate exertional capacity. Finally, enhanced sympathetic activity exaggerated by increased peripheral and central chemoreceptors (Ponikowski and Banasiak, 2001), with diminished function of baroreceptors (Ponikowski et al., 1997) and abnormal signalling from muscular ergoreceptors (Piepoli et al., 1996) modify the circulatory and ventilatory response to exercise. In our study, we focused on the role of chemoreflex (arising from carotid bodies) and tonic activity of PChRs in the ventilatory and hemodynamic control during exercise.

### Mechanisms of altered PChRs’ function in HFpEF

Our study population included mainly elderly women with preserved ejection fraction of the left ventricle and high prevalence of various co-morbidities typical for HFpEF (hypertension, obesity, atrial fibrillation) (Table 1). The study population was characterized by moderate to good quality of life and moderate worsening of physical endurance based on 6-minutes walking test and cardiopulmonary exercise test indices when measured on placebo.

Interestingly, the PChS in the studied HFpEF population was similar to that observed by Niewinski et al. in patients with HFrEF (0.63 L min^−1^ %^−1^ vs. 0.58 L min^−1^ %^−1^) (Niewinski et al., 2013). This is particularly surprising, since described HFrEF population clearly presented with more advanced disease (higher level of natriuretic peptides and NYHA class) comparing to currently investigated HFpEF cohort. We hypothesise that this finding could be explained by higher prevalence of the co-morbidities (especially obesity and impaired glucose metabolism) which may have additional impact on PChS.

Two-thirds of the study population suffered from diabetes mellitus or impaired glucose tolerance. There are some premises suggesting that PChRs contribute to the control of glucose metabolism (Ward, Voter and Karan, 2007; Wehrwein et al., 2010). Ribeiro et al. demonstrated augmented CBs activity in rats which developed insulin resistance and hypertension after 3-4 weeks of hypercaloric diet (Ribeiro et al., 2013). Importantly, bilateral CBs resection abolished these diet-induced disturbances.

Visceral obesity was also present in most patients investigated in our study. Obesity often coexists with obstructive sleep apnea syndrome where repetitive apnea/hypopnea episodes augment CBs sensitivity and increase sympathetic gain (Paton, Sobotka et al., 2013; Iturriaga, Andrade and Del Rio, 2014). Furthermore, obese patients are characterized by exaggerated production of leptin which leads to sympathoexcitation (Eikelis et al., 2003). Leptin and leptin receptors were observed in human and rat type I glomus cells within CBs (Porzionato et al., 2011) and intravenous injection of leptin resulted in CBs activation (Messenger, Moreau and Ciriello, 2012).

Moreover, all patients in our population suffered from hypertension which has been previously linked to PChRs’ oversensitivity (Prabhakar and Peng, 2004) and hypertonicity. The latter was elegantly showed by Sinski et al. who used 100% oxygen to block PChRs in a group of hypertensive males, what led to significant reduction in both blood pressure and muscle sympathetic nerve activity (Sinski et al., 2014). This was not observed in normotensive peers.

The interpretation of PChS level in our work is limited by the lack of control group of age- and sex-matched healthy individuals. In the previous studies, where identical methodology of PChS assessment was employed (Chua and Coats, 1995; Niewinski et al., 2013; Paleczny et al., 2014; Tubek et al., 2018) consistently lower levels of PChS had been reported in healthy controls (0.2-0.39 L min^−1^ %^−1^). However, all those groups were characterized by considerably lower age than currently studied HFpEF population.

### The effects of low-dose dopamine on resting parameters (tonicity)

In the current study we used dopamine in a dose of 3 μg kg^−1^ min^−1^ in order to activate selectively D_2_ presynaptic receptors (Niewinski and Tubek, 2014; Limberg et al., 2016) on glomus type I cells (Lehmann, Briley and Langer, 1983), that results in decreased release of neurotransmitters and thus leads to diminished discharge rate within sinus nerves (González et al., 1995). On the contrary, dopamine in high doses stimulates both pre-synaptic D_2_ and post-synaptic type 1 dopamine receptors (D_1_) resulting finally in the augmentation of the neural traffic from CBs with subsequent hyperventilation, due to relative domination of D_1_ receptors (Welsh, Heistad and Abboud, 1978; González et al., 1995).

We observed that dopamine administration reduced resting MV with a concomitant decrease in SpO_2_. These changes are solely related to the direct blockade of D_2_ receptors in PChRs since the blockade of β-receptors and stimulation of α-receptors does not influence this response (Welsh, Heistad and Abboud, 1978). Our results are concordant with previous studies where dopamine diminished normocapnic ventilation in patients with HFrEF, but not in age-matched healthy volunteers (Welsh, Heistad and Abboud, 1978; van de Borne, Oren and Somers, 1998). These findings underline the significant role of PChT in the breathing control in whole HF population. Another possible explanation by which dopamine might reduce MV is the effect on pulmonary J receptors. One could hypothesize that dopamine by increasing CO leads to lung decongestion and thus diminishes activation of pulmonary J receptors involved in hyperventilation in HF (van de Borne, Oren and Somers, 1998). We believe that such possibility is rather unlikely, as the time course of changes in MV (minutes) following dopamine introduction precludes significant decongestion.

Another important observation related to the initiation of low-dose dopamine was a fall in SVR. As previously shown low-dose dopamine leads to a decrease in blood pressure, systemic peripheral resistance and renal vascular resistance in an experimental animal model (Setler, Pendleton and Finlay, 1975). Dopamine in dogs with congestive HF provoked vasodilatory response and improved hindlimb flow and conductance at rest and during exercise (Stickland et al., 2007). Edgell et al. presented data indicating that dopamine attenuated ventilation and total peripheral resistance index at rest in HF with no impact on these parameters in healthy peers (Edgell et al., 2015).

The drop in SVR was observed also in studies employing hyperoxia as PChRs inhibitor—in both HFrEF (Tubek et al., 2021) and hypertensive patients (Sinski et al., 2014). Thus, it could be speculated that decline of SVR seen in our study was at least partially due to the inhibition of PChRs with subsequent reduction in sympathetic neural traffic to systemic vasculature. However, we cannot exclude direct vasodilatory effect related to the stimulation of D_2_ receptors within peripheral vessels (Overgaard and Džavík, 2008). This has to be confirmed in the future studies employing microneurography recordings.

We also reported an augmentation of HR and CO following dopamine infusion. One plausible explanation of this effect would be a direct stimulation of β_1_-receptors. Dopamine at medium doses (3-10 μg kg^−1^ min^−1^) shows an affinity to β_1_‐receptors which may lead to mild positive inotropic and chronotropic effects (Overgaard and Džavík, 2008). On the other hand, one could hypothesize that increase in CO and HR may be a compensatory mechanism (possibly baroreflex-mediated) in order to maintain adequate blood supply when SVR is falling. This is further supported by the fact that inhibition of PChRs results in improved baroreflex sensitivity as documented by Ponikowski et al. (Ponikowski et al., 1997).

### The effects of low-dose dopamine on acute peripheral chemoreflex sensitivity

Apart from resting parameters during normoxia (PChT), low-dose dopamine infusion also diminished the ventilatory response to hypoxia (PChS) and MAP slope, but did not influence HR slope when compared to placebo. This observations are in line with the results of our previous studies performed in healthy individuals (Niewinski and Tubek, 2014). The dissociation regarding the influence of low-dose dopamine on the particular components of hemodynamic response to acute PChRs stimulation may be explained by the dualism of HR response. The cardiovascular reflex from PChRs includes primary and secondary components (Paton, Ratcliffe et al., 2013). The primary response to hypoxia from CBs is a vagally-mediated bradycardia, while an excitation of ABs results in tachycardia (Daly and Scott, 1962; Tubek et al., 2016). The secondary response is initiated by hyperventilation, and subsequent activation of pulmonary stretch receptors (Hering-Breuer reflex), which promotes tachycardia (Kumar, 2009). Therefore, the final result of non-selective PChRs stimulation (tachycardia vs. bradycardia) depends on the strength of the ventilatory reactivity, and the predominance of the aforementioned primary vs. secondary mechanisms. We speculate that dopamine may inhibit both types of the responses. Thus, on the one hand primary responses arising from CBs and ABs become limited, and on the other hand the magnitude of Hering-Breuer reflex, which promotes tachycardia, is reduced (due to decreased hypoxic ventilatory reactivity) resulting in an unchanged HR response to hypoxia.

### The effects of low-dose dopamine on exercise tolerance

We showed that inhibition of PChRs with low-dose dopamine results in an improvement (decrease) of V˙E/V˙CO_2_ slope. The decline in V˙E/V˙CO_2_ was modest but still statistically significant. Our results are supported by the fact that dopamine infusion did also significantly decrease nadir V˙E/V˙CO_2_ (32 ± 3.4 vs. 30.8 ± 3.7, *p* = 0.03). Furthermore, our findings correspond well with previous papers reporting numerically modest but statistically significant improvements in ventilatory efficiency (reflected by V˙E/V˙CO_2_ slope) following various interventions. In the paper by Chua et al. blockade of peripheral chemoreceptors with dihydrocodeine led to decrease in V˙E/V˙CO_2_ slope from 34.19 ± 2.35 to 30.85 ± 1.91 (*p* = 0.01) (Chua, Harrington et al., 1997). Modulation of peripheral chemoreflex with cardiac resynchronization therapy (4-6 months after device implantation) led to decline in V˙E/V˙CO_2_ from 44 ± 10 to 40 ± 8, *p* < 0.01 (Cundrle et al., 2015). Treatment with carvedilol was also associated with numerically small difference in V˙E/V˙CO_2_ slope when compared to patients taking bisoprolol (29.7 ± 0.4 vs. 31.6 ± 0.5, *p* = 0.023) (Agostoni et al., 2010). Finally, initiation of sacubitril-valsartan was related to a decline in V˙E/V˙CO_2_ of only 2.4 (from 34.1 ± 6.3 to 31.7 ± 6.1; *p* = 0.006) as recently reported by Vitale et al. (Vitale et al., 2019).

V˙E/V˙CO_2_ is an index of ventilatory effectiveness on exertion. Higher level of this parameter reflects increased ventilatory drive and is a well-recognized marker of poor prognosis (Francis, 2000; Arena et al., 2008). Shen et al. demonstrated that V˙E/V˙CO_2_ slope ≥39.3 was related to increased cardiac mortality, whereas V˙E/V˙CO_2_ slope ≥32.9 was linked to elevated risk of cardiac-related hospitalizations in HFrEF patients (Shen et al., 2015). There are many factors responsible for elevated V˙E/V˙CO_2_ slope in the HF population: enlarged physiological (Kleber et al., 2000) and anatomical dead space (Buller and Poole-Wilson, 1990), impaired pulmonary vascular hemodynamics (Metra et al., 1992), ventilation-perfusion mismatch (Uren et al., 1993) and abnormal ventilatory reflex control (Chua, Clark et al., 1996; Ponikowski et al., 2001). The latter was demonstrated by Ponikowski et al. who showed that increased PChS is related to higher V˙E/V˙CO_2_ slope in congestive HF (r = 0.27, *p* = 0.015) (Ponikowski et al., 2001). Similar findings were reported by Giannoni et al. (r = 0.42, *p* < 0.001) (Giannoni et al., 2008).

Mathematically a decrease in V˙E/V˙CO_2_ slope could be a result of either diminished ventilation or increased production of CO_2_. As we did not find a significant difference in peak V˙CO_2_ between stress tests performed on placebo and dopamine, it should be assumed that the attenuation of ventilation during exercise is mostly responsible for this benefit. An inhibition of PChRs with dopamine diminishes ventilation at rest and we assume that this effect is also in operation during whole exercise. This is further supported by the fact that the degree of improvement (decrease) in V˙E/V˙CO_2_ slope was proportional to the magnitude of MV attenuation following dopamine initiation (PChT). A decrease in tonic respiratory drive from PChRs might be further aggravated by a concomitant decrease in central respiratory drive, as a hyperadditive interaction between PChRs and central chemoreceptors has been previously described (O’Regan and Majcherczyk, 1982; Gonzalez et al., 1994). Interestingly, in the study by Collins et al. dopamine administration (in similar dose of 2 μg kg^−1^ min^−1^) caused improvement in vascular conductance and oxygen delivery with no changes in ventilation (Collins et al., 2020). This difference may be explained by the divergent group of patients studied in the current paper (exclusively HFpEF vs. primarily HFrEF). Additionally, patients studied by Collins et al., presented with considerably better exercise tolerance and ventilatory efficiency at the baseline (reflected by mean peak V˙O_2_ of 25 ml kg^−1^ min^−1^ and V˙E/V˙CO_2_ of 31.7). Therefore, we speculate that due to possibly low PChT in this apparently mildly affected population low-dose dopamine could not exert its ventilatory benefits as seen in our study.

Reduction in V˙E/V˙CO_2_ slope has important clinical consequences. Attenuation of excessive ventilation on exertion reduces the subjective sensation of dyspnoea (Nakayama, 1962; Stulbarg and Winn, 1989), decreases the propensity for dynamic lung hyperinflation and potentially reduces the fatigue of respiratory muscles (O’Donnell et al., 1999). A rise in end-tidal CO_2_ and arterial CO_2_ partial pressure during exercise is another consequence of diminished ventilation following PChRs inhibition (Welsh, Heistad and Abboud, 1978; Chua and Harrington, 1997; van de Borne, Oren and Somers, 1998). As observed by Huckauf et al. with dopamine infusion (Huckauf, Ramdohr and Schröder, 1976) and following bilateral CBs resection (Niewinski et al., 2013) an increase in arterial CO_2_ partial pressure related to the blockade of PChRs is mild, and as such is unlikely to exert untoward effects from the clinical point of view. On the contrary, mild hypercapnia might reduce the risk for periodic breathing and thus for exercise oscillatory ventilation (Yajima et al., 1994), which is known to be related to poor exercise tolerance in HF (Schmid et al., 2008).

The unique protocol employed in our study allowed for the separate assessment of the involvement of PChS and PChT in the ventilatory efficiency during exercise. In the study by Chua (Chua, Ponikowski et al., 1996) inhibition of PChRs with oxygen prolonged exercise duration and improved ventilatory efficiency. In another study, administration of dihydrocodeine decreased hypoxic and hypercapnic ventilatory responses and improved exercise time, peak V˙O_2_ and V˙E/V˙CO_2_ slope (decrease from 34.19 ± 2.35 to 30.85 ± 1.91, *p* = 0.01) (Chua and Harrington, 1997). CB resection in HFrEF resulted in attenuation of PChS (Niewinski et al., 2017) and better exercise tolerance expressed as a prolongation of exercise duration and a fall in V˙E/V˙CO_2_ slope at 2 months (36.4 ± 3.0 vs. 32.8 ± 3.2, *p* = 0.03). It should be noted that in all mentioned studies (including our work) the improvement in V˙E/V˙CO_2_ occurred following the inhibition of both components of PChRs functionality.

Interestingly, we found that only PChT reflected the magnitude of improvement in exercise tolerance. In the study by Stickland et al. (Stickland et al., 2007) dopamine during mild-intensity exercise improved blood flow in the periphery (assessed as an increase in hindlimb conduction). Moreover, the vasodilatory effect observed after alpha-adrenergic blockade confirmed that sympathetic activation limited the blood flow in skeletal muscles. Interestingly, PChT was responsible for only one-third of the total sympathetic restraint in muscular blood flow pointing out other factors involved in cardiorespiratory control during exercise (such as central chemoreceptors, muscle ergoreceptors and baroreceptors) (Rowell and O’Leary, 1990).

On the other hand, a decrease in PChS following PChRs deactivation did not correlate with the relative (and absolute) reduction in V˙E/V˙CO_2_ slope. This can be explained by the following mechanisms. First, it is possible that the degree of inhibition of acute responsiveness arising from PChRs achieved with low-dose dopamine is not sufficient to translate into meaningful decrease in ventilation on exertion. Second, keeping in mind that the partial pressure of O_2_ remains fairly stable during exercise (Forster, Haouzi and Dempsey, 2012), it could be speculated that the inhibition of hypoxic response is not equal to the inhibition of the acute responsiveness to the metabolites of exertion (e.g. lactate). The latter explanation suggests that PChS is not directly involved in the ventilatory response to exercise in HF patients. If this is the case, than how should we interpret the positive relation between PChS and V˙E/V˙CO_2_ slope (during placebo infusions) seen in our and in numerous previous studies? We believe that high PChS ought to be seen more as a marker of advanced HF (in fact of the worse perfusion through CBs (Ding, Li and Schultz, 2011), where number of different factors could be contributing to the ineffective ventilation on exertion (e.g. pulmonary congestion, increased dead space, augmented sensitivity of metaboreceptors).

Although dopamine reduced V˙E/V˙CO_2_ slope in our study, it did not improve exercise tolerance as reflected by unchanged peak V˙O_2_. Thus, the exercise tolerance in HFpEF is likely determined by factors other than dysfunctional PChRs. For example, Toledo et al. demonstrated overactivation of central but not peripheral chemoreceptors in rats with HFpEF. Furthermore, acute stimulation of central chemoreceptors provoked increase in sympathetic gain, abnormalities in cardiac function and induced cardiac arrhythmogenesis (Toledo et al., 2017). Structural and functional muscular abnormalities must be also taken into consideration regarding limited exercise capacity in the HFpEF population (Haykowsky et al., 2014; Kitzman et al., 2014). In the study by Haykowsky et al. elderly patients with HFpEF were characterized by greater intermuscular fat area and higher ratio of intermuscular fat to skeletal muscle compared to age-matched healthy controls. Furthermore, both intrinsic muscle abnormalities were found to be predictors of low peak V˙O_2_ (Haykowsky et al., 2014).

The inhibition of PChRs presents as a promising approach for alleviating debilitating symptoms of HF. However, the safety aspects of such maneuver should be taken into consideration—especially the risk of profound hypoxemia due to diminished ventilatory responsiveness, which may be seen even during exposure to mild hypoxia (e.g. at high altitude or during long-haul air travels) (Niewinski et al., 2021). This aspect of PChRs modulation was not directly assessed in our study as the prolonged hypoxia was not a part of the experimental protocol.

Several study limitations should be acknowledged. First, the sample size was small, which precluded multivariable modeling, and was a likely reason of failure to evidence the significance of some associations. Due to small sample size our work should be seen as a pilot study. Thus, presented correlations ought to be interpreted with caution. Second, the majority of patients were women. In such population, we cannot exclude the plausible impact of sex hormones on PChRs function (Regensteiner et al., 1989; Prabhakar and Peng, 2004; Kumar and Prabhakar, 2012). In order to minimalize this effect, only postmenopausal women were enrolled into the study. Third, we did not have a control group, thus we cannot compare our result with the healthy peers. We should also emphasize, that PChS is only a putative determinant of PChRs’ sensitivity to the metabolites of exercise. We did not measure the lactate concentration during exercise which would definitely shed more light on the matter. Particularly, increase in lactate production in muscles during exercise was significantly faster in HF subjects compared to healthy controls (Scott et al., 2003). Further, we did not maintain isocapnic conditions when analyzing the PChRs response to acute hypoxia. Therefore, we cannot eliminate the effect of concomitant hypocapnia which might lead to underestimation of the reflex response (Keir, Duffin and Floras, 2020). However, the method used in the study, has been previously validated and accepted as a reliable tool in the assessment of PChS in human subjects (Chua and Coats, 1995; Niewinski et al., 2013), where it better simulates environmental or disease-related conditions, and was further found to have prognostic significance (Ponikowski et al., 2001). Also we did not take into consideration the inter-individual variability in the ideal dose of IV dopamine (Limberg et al., 2016). Establishing the most effective dose of dopamine for each individual subject would have been an optimal approach, however it would considerably prolong already busy study protocol. Nonetheless, a dose of 3 μg kg^−1^ min^−1^ employed in our work has been shown to effectively inhibit both acute and tonic activity of PChRs (Limberg et al., 2016).

Finally, our study was not a clinical trial and the power calculation was not conducted. However, as an exploratory physiological experiment, we rather intended to reveal the potential mechanisms involved in exercise intolerance in the patients with HFpEF. Further studies incorporating considerably larger populations are needed to fully address the effect of PChRs inhibition on the clinical end-points.

## Conclusion

This study demonstrated an exaggerated PChRs response to hypoxia (PChS) in HFpEF, the magnitude of which was similar to that previously reported in HFrEF (Niewinski et al., 2013). Our protocol allowed for the identification of two different determinants of the PChRs functionality: while PChS tended to correlate with worse exercise tolerance in basic conditions, the degree of exercise capacity improvement following the inhibition of PChRs with dopamine was associated only with PChT. Confirmation of our results in larger clinical trials, would give opportunity to modulate CBs in HFpEF patients. The profile of eligibility for this kind of treatment (by means of CBs denervation or pharmacological modulation of PChRs) should consider the level of PChT rather than the magnitude of PChS.

## Data Availability

The raw data supporting the conclusion of this article will be made available by the authors, without undue reservation.
